# Volume of Care for Primary Care Physicians in Integrated vs Independent Practices Through the COVID-19 Pandemic

**DOI:** 10.1001/jamahealthforum.2023.2883

**Published:** 2023-09-01

**Authors:** Alison Cuellar, Anupam B. Jena

**Affiliations:** 1 Department of Health Administration and Policy, George Mason University, Fairfax, Virginia; 2 National Bureau of Economic Research, Cambridge, Massachusetts; 3 Department of Health Care Policy, Harvard Medical School, Boston, Massachusetts; 4 Massachusetts General Hospital, Boston

## Abstract

This cohort study examines changes in volume of in-person vs virtual visits to independent and integrated practices during the COVID-19 pandemic.

## Introduction

Although the COVID-19 pandemic has had a disparate effect on physician practices, the mediating role of financial integration is largely unknown. In particular, vertically integrated practices (ie, those affiliated with a larger health care system) may have been able to draw on greater resources to maintain care vs independent practices. Previous studies of vertically integrated practices have not addressed the effect of large-scale events like the COVID-19 pandemic,^1^ and studies of practices during the pandemic are limited by reporting bias from convenience samples.^2^ We examined changes to in-person and virtual care during the pandemic for patients treated by primary care physicians (PCPs) in independent vs vertically integrated practices and examined subgroups of patients by chronic conditions.

## Methods

This cohort study used claims data from FAIR Health, a national database covering approximately 75% of the privately insured population in the US. We limited the analysis to a balanced panel of PCPs with 25 or more attributed patients in the past year with asthma, depression, hyperlipidemia, hypertension, no chronic conditions, and multiple chronic conditions.^3^ We obtained quarterly counts of office and telehealth visits for patients aged 18 to 64 years from March 1, 2018, through June 30, 2021. We identified physicians in the same practice as integrated based on evaluation and management visits and place of service^4^ (eMethods in Supplement 1). The George Mason University institutional review board determined this study exempt as data were deidentified. We followed the STROBE reporting guideline.

We estimated physician-level, quarterly, multivariable event study models (relative to quarter in which the public health emergency was issued) for total office visits and telehealth visit proportions. We included physician fixed effects to control for time-invariant characteristics, including sex, age, and geographic area. Data on race and ethnicity were not available. We adjusted for median social distancing using county-level SafeGraph data to adjust for the time-varying impact of local closures on level of area movement.^5,6^ Physician geography was defined by 493 areas, corresponding approximately to 3-digit zip codes, which were mapped to counties for aggregation (eMethods in Supplement 1). Analyses were performed using Stata, version 16 software (StataCorp LLC).

## Results

The sample included 4596 physicians in integrated practices (1961 graduated before 1990 [42.7%]; 2224 women [48.4%] and 2372 men [51.6%]) and 32 316 in independent practices (18 190 graduated before 1990 [56.3%], 13 797 women [37.4%], 23 115 men [62.6%]). Our panel ranged from 186 956 (patients with depression) to 516 768 (patients with no chronic conditions) physician-quarter observations. The Table shows descriptive baseline differences. Overall, physicians in integrated practices experienced reductions in volume for patients with no chronic conditions relative to independent practices in the first postpandemic quarter (−18.75%; 95% CI, −25.95% to −11.55%), decreasing to −14.05% (95% CI, −19.95% to −8.15%) in the sixth postpandemic quarter (Figure, A). Patterns for other conditions were similar. In most cases, differences in volume became smaller with time. In contrast, physicians in integrated practices experienced increases in the proportion of telehealth visits for individuals with no chronic conditions vs independent practices in the first postpandemic quarter (7.59%, 95% CI, 6.08%-9.10%); similar differences were found for all subgroups (Figure, B).

**Table.** Baseline Outcomes and Region for Primary Care Physicians in Integrated and Independent Practices

	No chronic conditions		Multiple chronic conditions	
	Integrated practice	Independent practice	Integrated practice	Independent practice
Total patients with visits, mean (SD)	12.80 (18.95)	10.93 (16.49)	20.98 (26.80)	19.05 (24.52)
Proportion telehealth visits, mean (SD)	0.30 (0.40)	0.24 (0.38)	0.37 (0.42)	0.31 (0.42)
Median area social distancing, mean (SD)	91.75 (6.32)	90.44 (6.47)	91.70 (6.33)	91.40 (6.49)
Region, No. (%)				
Midwest	713 (15.6)	4295 (13.3)	658 (15.6)	4065 (13.2)
Northeast	2284 (49.7)	10 914 (33.8)	2152 (50.9)	10 494 (34.2)
South	367 (8.0)	6954 (21.5)	320 (7.6)	6603 (21.5)
West	1232 (26.8)	10 153 (31.4)	1095 (25.9)	9537 (31.0)
No. of PCPs	4596	32 316	4225	30 699

Abbreviation: PCP, primary care physician.

**Figure.**
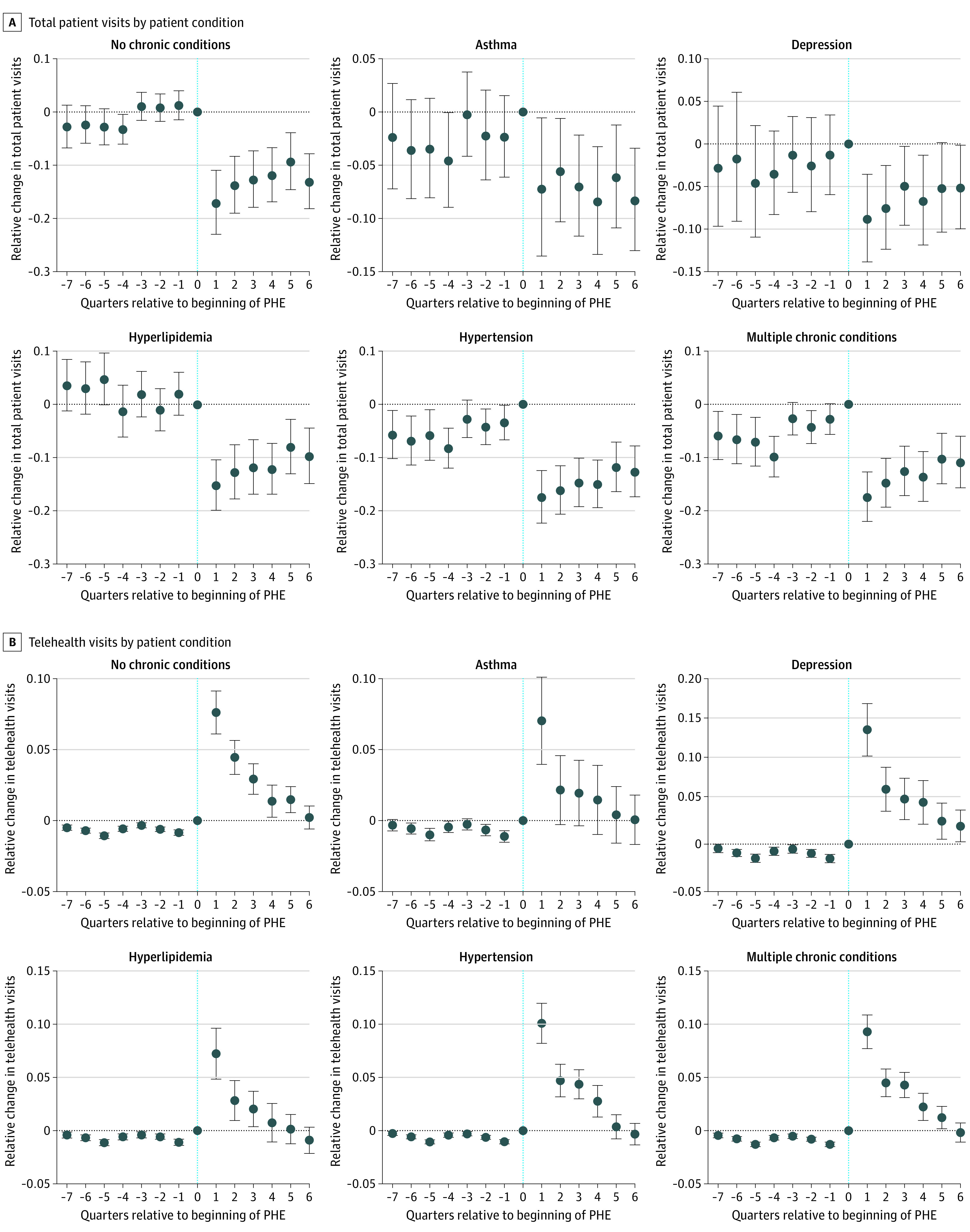
Event-Time Estimates by Patient Condition and Primary Care Physicians in Integrated vs Independent Practices Estimates are reported, with whiskers indicating 95% CIs. Each estimate summarizes the association between the beginning of the public health emergency (PHE) and the relative difference in outcomes between vertically integrated and independent practices at each point in time relative to the PHE. The dotted vertical line marks March 1 to May 31, 2020. A, Coefficients and 95% CIs transformed to semielasticites [exp(b) − 1]. Poisson regressions were used to control for physician fixed effects and median area social distancing. B, Linear regressions were used to control for physician fixed effects and median area social distancing.

## Discussion

The findings show that PCPs in integrated practices saw relatively fewer total patients vs those in independent practices in the first 18 months of the COVID-19 pandemic, suggesting that they may have been under less financial pressure to maintain patient volume. Despite overall reductions in patient volume vs independent practices, PCPs in integrated practices were more likely to see patients via telehealth, consistent with greater resources, faster adoption of health technology, and differences in practice culture or geography. Study limitations included the use of private claims data, which may lack relevant clinical information; unobserved differences in patient case mix; and the observational nature of the study, all of which preclude a causal interpretation of the findings.

## Notes

Supplement 1.
eMethods.

Supplement 2.
Data Sharing Statement
