# Supplementary material for: Volume of Care for Primary Care Physicians in Integrated vs Independent Practices Through the COVID-19 Pandemic
Source: JAMA Health Forum. 2023 Sep 1;4(9):e232883. doi: 10.1001/jamahealthforum.2023.2883 (PMC10474525; doi:10.1001/jamahealthforum.2023.2883)
Supplement: Supplement 1. — eMethods. [file jamahealthforum-e232883-s001.pdf]

Supplemental Online Content

Cuellar A, Jena AB. Volume of care for primary care physicians in integrated vs independent practices through the COVID-19 pandemic. *JAMA Health Forum*. 2023;4(9):e232883. doi:10.1001/jamahealthforum.2023.2883

eMethods.

This supplemental material has been provided by the authors to give readers additional information about their work.

© 2023 Cuellar A et al. *JAMA Health Forum.*

eMethods

Attribution:

In order to attribute a member to a primary physician who is responsible for the patient with the condition, the following methodology was used. First, identify the following Evaluation and Management services:
• _99201 – 99215 – Office or Other Outpatient Service
• _99381 – 99429 – Preventative Medicine Visits
• _99241 – 99255 – Consultations
• _90791 – 90899 – Psychiatric and Psychotherapy Visits*

* These codes were only used for patients who have a behavioral health condition:
 ADHD, Conduct Disorders, and Hyperkinetic Syndrome
 Bipolar Disorder
 Depressive Disorders
 Schizophrenia
 Substance Use Disorder

with a date of service in the prior one year from the year of analysis performed for the patient on the claim line. If any of the procedure codes or diagnosis codes appear on a claim line with a date of service in the one-year window being evaluated, then the applicable member identifier and Provider NPI combination are flagged for use. Providers with a Hospital, Urgent Care, Radiology or Laboratory taxonomy and/or specialty are not included.

An example would be that data would be evaluated for March 1, 2017 through February 28, 2018 to attribute a provider to a patient, and in 2018 (i.e., March 1, 2018 through February 28, 2019) that patient would then be attributed to the provider for that year. For the analysis only primary care physicians were included.

Chronic Conditions:

Chronic Conditions were defined using the Medicare Chronic Conditions Warehouse definitions (https://www2.ccwdata.org/web/guest/condition-categories). FAIR Health identified patients with chronic conditions (asthma, depression, hypertension, hyperlipidemia, multiple chronic conditions, and none) and limited the analysis to patients who had the relevant condition on at least two claims.

Estimation Equation:

The event-time model is estimated via the following equation,

$$Y_{pt}= \sum^{T}_{i=t} \beta_i \,(\theta_t \times D_p) + \theta_p + \theta_t + \lambda_{at} \text{ social distancing} + \varepsilon_{pt}$$

Where i indexes quarters relative to the COVID-19 pandemic start date, so i=0 is March 1 through May 31, 2020, i=1 is the quarter after the pandemic begins, while i=-1 is the quarter

© 2023 Cuellar A et al. *JAMA Health Forum.*

before the pandemic begins and so forth. θ_p is a physician fixed effect while θ_t is a vector of quarter indicators, where the pandemic start quarter is the omitted category. Social distancing is the time-variant measure of social distancing. Y represents the respective dependent variables (total visits or the proportion of visits via telehealth) by physician and quarter. D is a dummy variable indicating that a physician is in an integrated practice and zero otherwise and is interacted with each quarter indicator.

Geographic Areas:
Fairhealth defines 493 geozip regions which correspond in most cases to 3-digit zipcodes areas. The exceptions are the 3-digit zipcodes listed below which are combined. For the descriptive table 3 digit zipcodes were mapped to states using ZIP Code Tabulation Area (ZCTA) Relationship files: https://www.census.gov/geographies/reference-files/2010/geo/relationship-files.html#par_textimage_674173622, and then mapped to Census regions.

013 and 014
021 and 022
030, 031, and 033
032, 034 and 036
039 and 040
043, 045, and 049
044, 046, and 048
050, 052, 053, 056, 057, 058, and 059
062 and 063
068 and 069
070 and 072
071 and 073
074 and 075
080 and 084
100, 101, 102
107, 108
111 and 112
113, 114
115, 116
117, 119
120 and 121
124, 127
129, and 136
130 and 131
133, 134, 135
137, 138, and 139
140, 141, and 143
144 and 145
148 and 149
150 and 151
153 and 154
155, 156, and 15
158, and 162
163 and 167
164 and 165
169, 177, and 178
170, and 171
173 and 174
179 and 181
180 and 181
184 and 185
186 and 187
195 and 196
197 and 198
200, 202, and 205
208 and 209
210, 211, and 219
215 and 217
216 and 218
222 and 223
224, and 245
226 through 229
230, 231, and 238
233 through 237
239, 244 and 245
240 and 241
242, 243, 246
247 through 249, 258, 259, and 266
250, 252, 253, 255, 256 and 257
254, 261, 264, 267, 268
260 and 265
270 and 273
278, 279, 285
280, 281

© 2023 Cuellar A et al. *JAMA Health Forum.*

282, 284
287, 288, 289
290, 291
298, 299
301, 302
303, 311
304, 315
308-309
313, 314
316 through 319, 398
323, 324, 325
350, 351, 354, 355, 356
357, 358
359, 362
360, 361, 364, 367
363, 368
365, 366, 369
370, 371
377, 378, 385
382, 383
387, 389, 391, 393
388, 397
400, 401,
403, 404
405, 406
407-409, 425-427
411-418
420-424
430-431
434 and 435
437-439, 457
442, 443
444, 445
446, 447
448, 449
450, 451
453, 455
460, 461, 473
463, 464
467-468
469, 479
470-472, 474, 478
475-477
484-485
486-487
488-489
490-492
493-494
496-497
498-499
500-503
504-505, 513
506, 507, 521
508, 510, 512, 514, 516
522, 523, 525, 526
527, 528
535, 538
541, 542
545, 548
556, 557
561-562
563, 564
565-567
570, 572, 577
580, 582, 583
585, 586
590, 592, 597, 599
602-603,
606, 608
613, 615
624, 628
625, 626
635, 644, 646
636, 637
638, 639
649, 656, 657
650, 651
654, 655
664, 665
667, 668, 673
669, 674, 676, 679
670, 671, 675
680, 687
683, 684, 686, 688, 693
703, 705, 706
710-714
716-719, 728
720-721
723-726
727, 729
734-736, 745, 747, 749
737-739, 746

© 2023 Cuellar A et al. *JAMA Health Forum.*

743, 744
752, 753
754, 755
758, 759
762, 763
764, 785, 796
765, 778
766, 767
768, 769, 788, 797
770, 772
776-777
779, 789
783-784
790, 792
793, 794
798, 799
806-807
808-809
810-815
820-831
832, 834
833, 835, 836, 838
843-844
845, 847
851, 852
855, 859, 863, 864
856, 857
860, 865
870, 873, 875, 877, 884
878-883
890-891
893-895, 897-898
900-901
902-903
910, 912
911, 918
915, 916
922-924
932, 933, 935
936, 937
974-977
978-979
983-984
988-989, 993, 994
990-992

© 2023 Cuellar A et al. *JAMA Health Forum.*
