# Supplementary material for: Volume of Care for Primary Care Physicians in Integrated vs Independent Practices Through the COVID-19 Pandemic
Source: JAMA Health Forum. 2023 Sep 1;4(9):e232883. doi: 10.1001/jamahealthforum.2023.2883 (PMC10474525; doi:10.1001/jamahealthforum.2023.2883)
Supplement: Supplement 2. — Data Sharing Statement [file jamahealthforum-e232883-s002.pdf]

Data Sharing Statement

Cuellar. Volume of Care for Primary Care Physicians in Integrated vs Independent Practices Through the COVID-19 Pandemic. *JAMA Health Forum*. Published September 01, 2023. doi:10.1001/jamahealthforum.2023.2883

Data
Data available: No

Additional Information
Explanation for why data not available: The data can be accessed via a Data Use Agreement directly with FAIRhealth, but are not available from the authors.
